# Transcriptome Reveals the Differential Regulation of Sugar Metabolism to Saline–Alkali Stress in Different Resistant Oats

**DOI:** 10.3390/genes16010105

**Published:** 2025-01-20

**Authors:** Naiyu Chen, Shuya Xing, Jiaxin Song, Shutong Lu, Lei Ling, Lina Qu

**Affiliations:** 1 Heilongjiang Provincial Key Laboratory of Oilfield Applied Chemistry and Technology, Daqing 163712, China; 2 College of Bioengineering, Daqing Normal University, Daqing 163712, China

**Keywords:** saline-alkali stress, oats, soluble sugars, carbohydrate metabolism, transcriptomics, abiotic stress

## Abstract

Background: Saline–alkali stress is a major factor limiting the growth of oats. Sugar is the primary carbon and energy source in plants which regulates plant development and growth by regulating enzyme activity and gene expression. Sucrose, glucose, and fructose are ubiquitous plant-soluble sugars that act as signalling molecules in the transcriptional regulation of various metabolic and defence-related genes. Methods: In this study, soluble sugars, fructose, sucrose, and starch contents were measured, and transcriptomics was used to determine the differentially expressed genes (DEGs) in saline-sensitive and saline-tolerant oats after 6, 12, 24, and 48 h. DEGs annotated to carbohydrates were selected using the Kyoto Encyclopedia of Genes and Genomes. Results: DEGs involved in carbohydrate metabolism were mainly enriched in the glycolysis/gluconeogenesis and pentose phosphate pathways, fructose and mannose metabolism, and starch and sucrose metabolism. *GAPDH*, *SUPI*, *SUS2*, *ATP-PEK*, *HXK6*, *FBA4*, *TBA4*, *TKT*, *ISA3*, *PPDK1*, and *BAM2* were significantly expressed, and a quantitative reverse transcription polymerase chain reaction verified the transcriptome sequencing results. Conclusions: In this study, oats with different salinity tolerances were used to determine sugar contents under four salinity stress durations, and transcriptome sequencing was used to explore the regulatory mechanism of sugars and provide a reference for elucidating the sugar signalling regulatory mechanism under abiotic stress.

## 1. Introduction

The global saline–alkali soil area exceeds 833 million hm^2^, accounting for 8.7% of the total area of the earth and covering 85% of the global land area, with more than 42.40 billion hm^2^ of topsoil and 3.01 billion hm^2^ of subsoil affected by salt damage [1]. The global population will face major challenges in food production owing to soil degradation. The area of saline–alkali land in China is 99.13 million hm^2^, ranking third worldwide, accounting for 1/10 of the total saline–alkali land area worldwide [2]. Therefore, there is an urgent need to control the salt and alkali issue. Globally, oats (*Avena sativa* L.) are the sixth most productive grain crop [3] owing to their nutritional value for human consumption, animal feed, healthcare, and cosmetics [4]. Moreover, oats exhibit a higher salt tolerance than rice, wheat, and other feed crops. Therefore, oats can be used as pioneer crops to improve saline soils [5].

Sugars are the main source of carbon and energy in plants. They also act as key signalling molecules that regulate plant development and growth by regulating enzyme activity and gene expression [6]. Soluble sugars play a key role in scavenging reactive oxygen species and mediating cold tolerance [7]. Saccharose [6], amylaceum [8], and fructose [9] are ubiquitous plant-soluble sugars that act as signalling molecules in the transcriptional regulation of various metabolic and defence-related genes. Sugars respond positively to abiotic stress and root cell wall polysaccharides play a crucial role in the response to waterlogging stress in *Brassica napus* cultivars [10]. A shortage in the carbohydrate supply in anthers leads to a severe loss of seed setting in rice under cold stress [11].

Transcriptome analysis is a powerful technique for determining regulatory networks triggered in plants under stress conditions. It is widely used to identify specific genes and genetic components that respond to stress [12]. Many genes that respond to salt stress have been identified in various crops, including sorghum [13], paddy [14], soybean [15], and rape [16].

Combined analysis of the transcriptomic metabolism of oat varieties with different salt tolerances revealed elevated levels of most sugars and amino acids in both varieties [17]. Oolong tea leaves exhibit increased levels of most sugar metabolites under abiotic stress, and most genes associated with sugar hydrolysis are downregulated [18]. Rapeseed differentially expressed genes (DEGs) were enriched in glycolysis/gluconeogenesis, amino sugar and nucleotide glucose metabolism, galactose metabolism, pentose and glucuronide interconversion, pentose phosphate pathway, and starch and sucrose metabolism [19]. The genes of the salt-tolerant oat BY 2 were enhanced in glycolysis and starch sucrose metabolism pathways under salt stress [17]. The aforementioned research indicates that carbohydrate metabolism plays a positive role in the stress response process of plants to abiotic stress. However, there is a scarcity of in-depth research on the regulatory mechanism of carbohydrate metabolism in oats under salt–alkali stress.

In this study, two oat varieties with different tolerances to saline–alkali stress were prepared, the soluble sugar, fructose, sucrose, and starch contents were measured, and transcriptome sequencing analysis was conducted. Four significantly different pathways related to glucose metabolism were selected for further analysis, and the DEGs were verified using a reverse transcription quantitative polymerase chain reaction (RT-qPCR). This study combined physiological and transcriptomic analyses to explore the differential genes and regulatory mechanisms of glucose metabolism in oats, before and after exposure to salinity stress. Our findings contribute to the understanding of glucose metabolism regulation under abiotic stress, with the aim of developing salt-tolerant cultivars to improve crop quality and yield.

## 2. Materials and Methods

### 2.1. Plant Materials and Salt–Alkali Stress Treatment

This experiment used four-week-old salt- and alkali-resistant oats, ‘Qinqiong’, and salt- and alkali-sensitive oats, ‘Menglong’. Single plants were planted using pot cultivation, and simulated salt–alkali stress treatment was conducted using Na_2_CO_3_ and NaHCO_3_ in a ratio of 1:1. The plants were independently subjected to salt–alkali stress for up to 6, 12, 24, and 48 h, and the control (CK) was normal oats without salt–alkali stress; there were three biological replicates per group.

### 2.2. Sugar Content Determination

The soluble sugar and starch contents were determined via anthrone colorimetry [20]. Sucrose and fructose contents were determined using the dinitrosalicylic acid and resorcinol methods [21].

### 2.3. RNA Extraction and Library Sequencing

Eukaryotic mRNA sequencing was conducted on the Illumina Novaseq 6000 sequencing platform, which sequences all mRNA transcribed from specific tissues or cells of eukaryotic organisms at a certain stage. The sequencing experiment uses the Truseq^TM^ RNA sample prep Kit (Illumina, San Diego, CA, USA) for library construction.

### 2.4. Sequencing Read Length Analysis and Splicing

Clean reads were mapped to the reference genome (Avena_sativa: reference genome version OT3098) using HISAT2 (Version 2.1.0). The transcript abundance of each gene was estimated (in FPKM), and the read count of each gene was obtained using RSEM (Version 1.3.3). The complete transcriptome analysis of 15 samples yielded a total of 98.77 Gb clean data. The clean data of each sample reached 6.08 Gb or above, and the Q30 base percentage was more than 94.56%. Sequence alignment was performed on the clean reads of each sample and the designated reference genome, with alignment rates ranging from 60.45% to 94.53%.

### 2.5. DEG Analysis

Based on the quantitative results of expression levels, differential gene analysis was performed between groups to identify the DEGs between the two groups. DESeq2 (Version 1.24.0) was used as the differential analysis software, and the screening threshold was log2|FC| ≥ 1 and *p* adjust < 0.05. All full-length transcripts were annotated using seven online libraries, namely the NCBI non-redundant protein database, Cluster of Orthologous Groups of proteins, Swiss-Prot Knowledgebase, Kyoto Encyclopedia of Genes and Genomes (KEGG), Eukaryotic Orthologous Groups (KOG), Gene Ontology (GO), and Protein Family (Pfam) databases.

### 2.6. RT-qPCR Analysis

Ten randomly selected genes were annotated to GO or KEGG. Their RNA was used as a template and the RNA was reverse-transcribed into cDNA; thereafter, RT-qPCR analysis was performed. The primer sequences are shown in Table 1. *Actin* was used as the reference gene. Gene expression levels were calculated using the 2^−ΔΔCT^ method, and the experiments were repeated thrice.

## 3. Results

### 3.1. Determination of Sugar Levels

**Figure 1:**
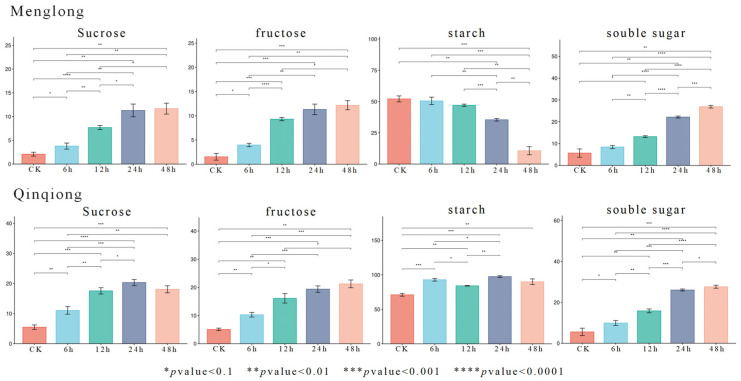
Sucrose, fructose, starch, and soluble sugar contents in Menglong (stress-sensitive) and Qinqiong (stress-tolerant) oats under four durations of salt–alkali stress.

The sucrose, fructose, starch, and soluble sugar contents of the saline-tolerant oat Qinqiong and saline-sensitive oat Menglong were determined. After salt–alkali stress, the sucrose content in Qinqiong (salt- and alkali-resistant oat) was more than twice that of Menglong (sensitive oat) before 48 h. However, at 48 h, the sucrose content in Qinqiong oats slightly decreased but was still substantially higher than that in Menglong oats. Under conditions of salt–alkali stress, the fructose content of both varieties of oats gradually increased; however, post-stress, the fructose content in Qinqiong oats was higher than that in Menglong oats. After 48 h of salt–alkali stress, the fructose content of the two oat varieties reached a maximum. At this time, the content of Qinqiong oats was 12 mg·g^−1^, and the content of Menglong was 19 mg·g^−1^. The most significant difference between the two varieties after salt–alkali stress was the change in the starch content. The starch content in Menglong oats gradually decreased, whereas it was relatively stable in Qinqiong oats. With the increase in the duration for which the oats were subjected to salt–alkali stress, the soluble sugar content of both varieties of oats gradually increased; however, at 48 h, the increase in the sugar content in response to salt–alkali stress in Menglong oats from 24 to 48 h was slightly greater than that in Qinqiong oats, but the content of soluble sugars in Qinqiong oats was still substantially greater than that in Menglong oats (Figure 1).

### 3.2. Data Quality Control

**Figure 2:**
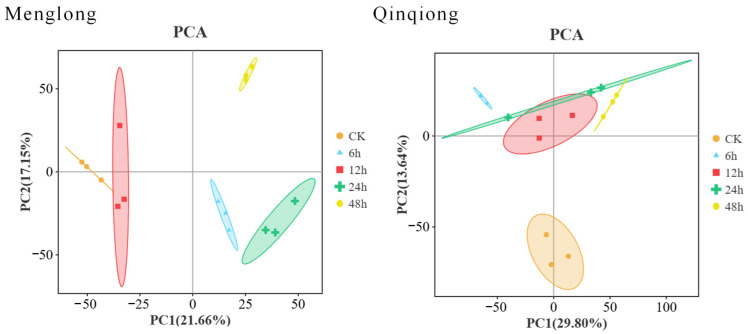
Principal components analysis (PCA) plots of sample quality control for Menglong (stress-sensitive) and Qinqiong (stress-tolerant) oats under four different durations of salt–alkali stress.

In the principal components analysis (PCA) of transcriptome data, the two primary principal components of the resistant varieties (Qinqiong) accounted for 29.80% and 13.64% of the differences, respectively, whereas the two primary principal components of the sensitive varieties (Menglong) accounted for 21.66% and 17.15% of the differences, respectively. The samples subjected to the same treatments clustered together, and substantial differentiation was observed between each treatment group, and between the oats after saline stress and the control. PCA based on the expression data revealed that 15 samples subjected to different processing times exhibited both repeatability and correlation (Figure 2). The high reliability of the subsequent analysis indicated that the overall sequencing quality of the RNA-seq reads was satisfactory and suitable for the subsequent correlation analysis.

### 3.3. Differential Gene Expression Analysis

**Figure 3:**
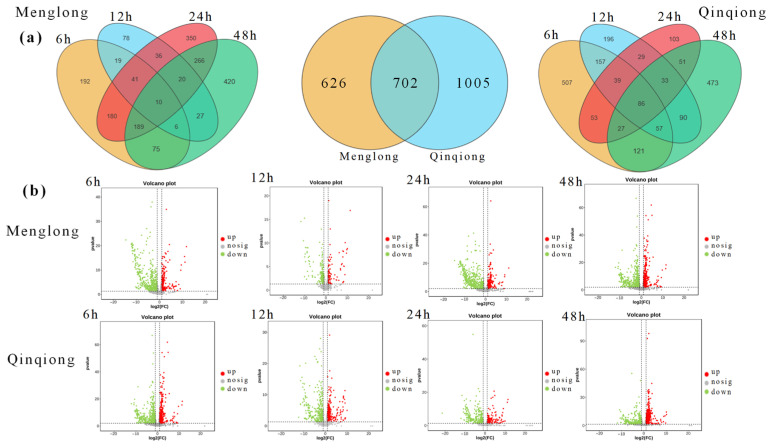
(**a**) From left to right, the Venn diagrams of significantly differentially expressed genes in Menglong oats (stress-sensitive) under four salt–alkali stress durations, significantly expressed genes in Menglong and Qinqiong (stress-tolerant) oats under at least one stress duration, and significantly differentially expressed genes in Qinqiong oats under four salt–alkali stress durations are shown. (**b**) The volcano plot shows significant differences in genes between Menglong and Qinqiong oats under four different durations of salt–alkali stress.

The screening criteria were established as *p* < 0.05, |fold change (FC)| ≥ 2, resulting in the identification of 1707 DEGs with significant expression in at least one period in the salt-resistant oat Qinqiong and 1328 in the salt-sensitive oats. A total of 702 DEGs were shared between the two varieties (Figure 3a).

Salt-tolerant oats exhibited 1047 DEGs after 6 h of salinity stress (upregulated: 432, downregulated: 615). Salt-sensitive oats had 712 DEGs during this period (upregulated: 291, downregulated: 421). At this time point, 197 DEGs were significantly expressed in both varieties of oats. There were 687 DEGs in salt-tolerant oats at 12 h (upregulated: 314, downregulated: 373). Salt-sensitive oats had 237 DEGs (upregulated: 71, downregulated: 166). At this time point, 52 genes were differentially expressed in both varieties of oats. At 24 h, salt-tolerant and -sensitive oats exhibited 421 (upregulated: 120, downregulated: 301) and 1092 (upregulated: 361, downregulated: 731) DEGs, respectively. At this time point, 85 genes were differentially expressed in both varieties of oats. At 48 h, the salt-tolerant oats had 939 DEGs (upregulated: 510, downregulated: 428). Salt-sensitive oats had 1013 DEGs (upregulated: 471, downregulated: 542) (Figure 3b). At this time point, 254 DEGs were significantly expressed in both varieties of oats. The DEGs in salt-tolerant oats before and after salinity stress exhibited a gradual decrease followed by an increase, whereas the DEGs in salt-sensitive oats demonstrated a substantial decrease followed by a significant increase.

### 3.4. Functional Annotation of DEGs

**Figure 4:**
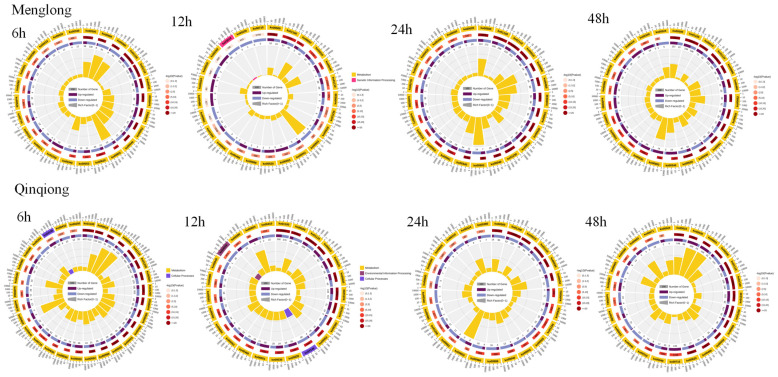
Enrichment circle diagram of differences in significant genes related to sugar metabolism in KEGG between Menglong (sensitive) and Qinqiong (tolerant) oats. The first circle represents the enriched classification, and the outer circle represents the coordinate scale of gene numbers. Different colours represent different classifications. Second circle: the number and *p*-value of the classification in the background genes. The more genes there are, the longer the bar, and the smaller the *p*-value, the redder the colour. Third circle: bar chart of the proportion of genes with upregulated and downregulated expressions, with purple and blue representing the number of genes with upregulated and downregulated expressions, respectively. The specific numerical values are displayed in the box. Fourth circle: RichFactor values for each category (the number of foreground genes divided by the number of background genes in that category).

The Kyoto Encyclopedia of Genes and Genomes analysis of the DEGs selected for related carbohydrates revealed that after 6 h of saline stress, the DEGs of salt-tolerant oats were primarily annotated to Ko00051 (fructose and mannose metabolism), Ko00030 (pentose phosphate pathway), and Ko00010 (glycolysis/gluconeogenesis). The main pathways enriched in the salt-sensitive oat DEGs were Ko009039 (limonene and pinene degradation), Ko00999 (starch and sucrose metabolism), and Ko00460 (cyanoamino acid metabolism). After 12 h of saline–alkali stress, the pathway with the most annotated DEGs in both salt-tolerant and -sensitive oats was Ko00603 (glycosphingolipid biosynthesis). After 24 h of saline–alkali stress, the main pathway in salt-tolerant oats was Ko00603, and the salt-sensitive oat pathways were Ko009039, Ko00999, and Ko00460. At 48 h of salinity stress, the most significant pathways in saline-resistant oats were Ko00051 and Ko00030. The main pathways enriched in the saline-sensitive oat DEGs were Ko00903 (limonene degradation), Ko00340 (histidine metabolism), and Ko00052 (galactose metabolism) (Figure 4).

### 3.5. Main Pathways of Glucose Metabolism

**Figure 5:**
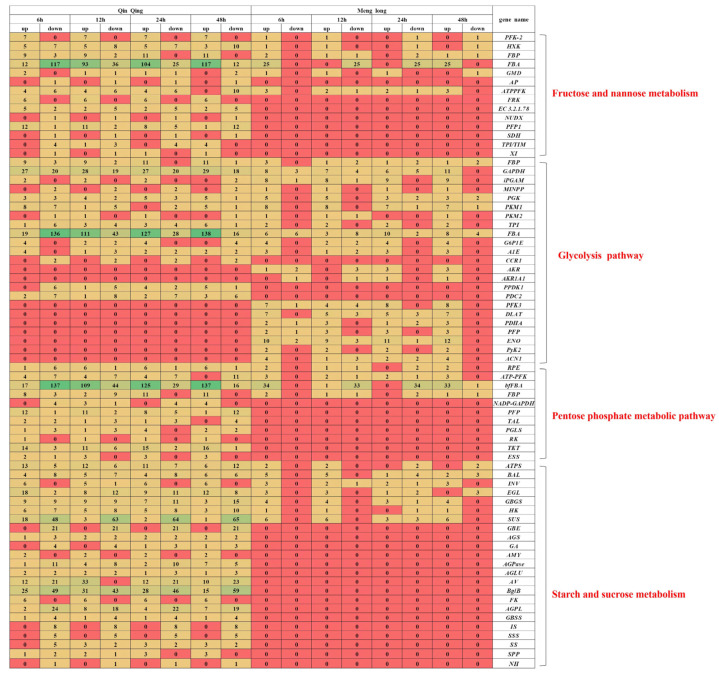
Heatmap showing the number of genes with upregulated and downregulated expressions in fructose, mannose, starch, and sucrose metabolism, glycolysis, and the pentose phosphate pathway in two oat varieties under four different durations of salt–alkali stress. The darker the colour, the more genes there are; the red colour indicates that there are no significantly different genes.

Using *p* < 0.05, |FC| ≥ 2 as the analysis criteria, examination of the DEGs in the significantly differential pathways of glycolysis, pentose phosphate metabolism, starch and sucrose metabolism, and fructose and mannose metabolism revealed that the tolerant type exhibited higher expression in the following pathways compared to the sensitive type: fructose and mannose metabolism (*PFK-2*, *HXK*, *FBP*, *FBA*, *GMD*, and *ATP-PFK*), pentose phosphate metabolic pathway (*RPE*, *ATP-PFK*, *bfFBA*, and *FBP*), and starch and sucrose metabolism (*ATPS*, *BAL*, *INV*, *EGL*, *GBGS*, and *HKSUS*). The sensitive type demonstrated higher expression than the tolerant type in the glycolysis pathway (*FBP*, *GAPDH*, *iPGAM*, *MINPP*, *PGK*, *PK1*, *PK2*, *TPI*, *FBA*, *G6P1E*, and *A1E*). Pathways with unique tolerant type expression included fructose and mannose metabolism (*AP*, *FRK*, *EC 3.2.1.78*, *NUDX*, *PFP1*, *SDH*, *TPI*, and *XI*), and the glycolysis pathway (*CCR1*, *PPDK1*, and *PDC*). Pathways with unique sensitive type expression included the glycolysis pathway (*AKR*, *AKR1A1*, *PFK-3*, *DLAT*, *PDHA*, *AKR*, *PFP*, *ENO*, *PyK2*, and *ACN1*), pentose phosphate metabolic pathway (*NADP-GAPDH*, *PFP*, *TAL*, *PGLS*, *RK*, *TKT*, and *ESS*), and starch and sucrose metabolism (*GBE*, *AGS*, *GA*, *AMY*, *AGPase*, *AGLU*, *AV*, *BglB*, *FK*, *AGPL*, *GBSS*, *Is*, *SSS*, *SS*, *SPP*, and *NH*) (Figure 5).

### 3.6. Gene Expression in the Carbohydrate Pathway

**Figure 6:**
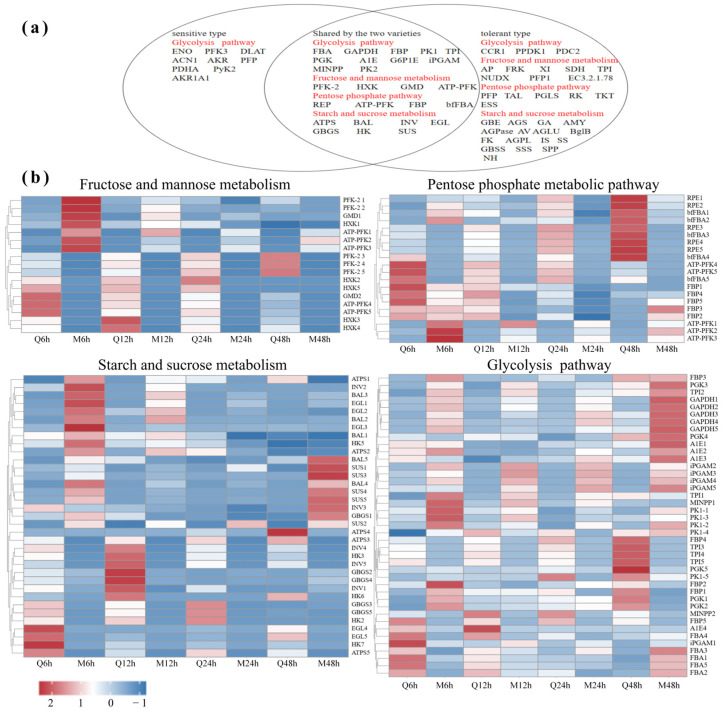
(**a**) Venn diagram showing significant differences in gene expression between the two varieties in terms of fructose, mannose, starch, and sucrose metabolism, the pentose phosphate pathway, and glycolysis. (**b**) Heatmap analysis of genes with significant differences between the two varieties in (**a**).

Using *p* < 0.05, |FC| ≥ 2 as the screening criteria, we have created a Venn diagram to visualise the differential genes associated with four significant sugar-related pathways (Figure 6a), and we have also generated a heatmap for the differentially expressed genes that are significantly shared between the two varieties (Figure 6b). The results reveal that in starch and sucrose metabolism under saline–alkali stress treatment, the differential expression ratio of the tolerant type was higher than that of the sensitive type for *HK*, *ATPS*, *INV*, and *GBGS*. The differential expression ratio of the sensitive type was higher than that of the tolerant type for *SUS* and *EGL*. Notably, the maximum upregulation multiple of *SUS* was significantly higher in the sensitive type at 12 h. In fructose and mannose metabolism under saline–alkali stress treatment, the differential expression ratio was higher in the tolerant type for *HXK*, whereas *PFK-2*, *ATP-PFK*, and *GMD* were higher in the sensitive type. Among them, the maximum upregulation multiple of *PFK-2* was significantly higher in the sensitive type at 6 h. In the pentose phosphate pathway under saline–alkali stress treatment, the differential expression ratio was higher in the sensitive type for *RPE*, *bfFBA*, and *FBP*. Among them, *RPE* and *bfFBA* were significantly higher in the sensitive type at 48 h, and *ATP-PFK* was higher than in the tolerant type. In the glycolytic pathway under saline–alkali stress treatment, the tolerant type’s differential expression ratios were higher than those in the sensitive type for *PGK*, *TPI*, *PK*, *GAPDH*, *FBP*, *A1E*, and *iPGAM*. Among them, the maximum upregulation ratio of *GAPDH* was significantly higher for the sensitive type. The differential expression ratio of the sensitive type was higher than that in the tolerant type for *MINPP* and *FBA*.

### 3.7. RT-qPCR

**Figure 7:**
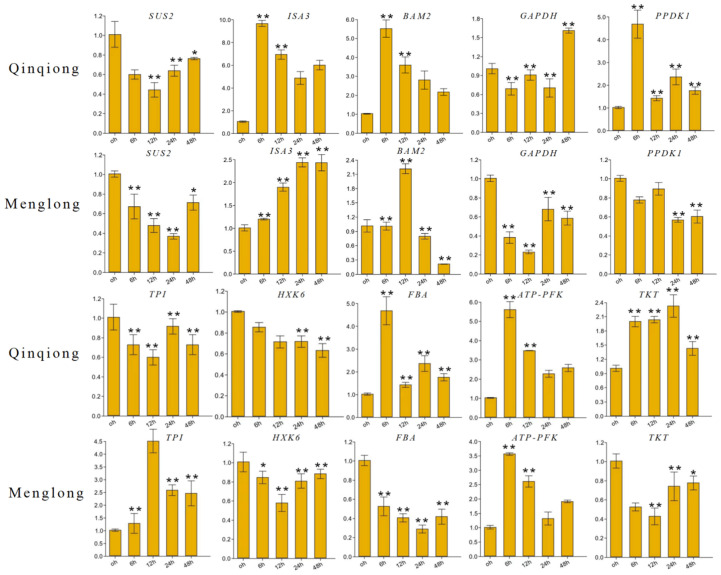
RT-qPCR analysis under four salt–alkali stress durations. Ten salt–alkali stress response genes were selected for RT-qPCR experiments. (* *p* < 0.05; ** *p* < 0.01) The *X*-axis represents the duration of salt–alkali stress treatment. The *Y*-axis represents the relative expression level of genes.

To investigate the expression patterns of related genes, RT-qPCR was used to analyse the expression of 10 key DEGs under conditions of saline–alkali stress. *SUS2* which plays a role in sucrose synthesis was downregulated in Menglong as well as Qinqiong oats, whereas *ISA3* and *BAM2* were upregulated in the salt-tolerant oats. *ISA3* expression gradually increased, whereas *BAM2* expression decreased after exhibiting the most pronounced upregulation at 12 h in the salt-sensitive type. *GAPDH*, which is involved in glycolysis, exhibited the most pronounced upregulation at 48 h in the salt-tolerant –type of oat but downregulation in the salt-sensitive type. *PPDK1* was upregulated in the salt-tolerant type of oat, whereas it was downregulated in the salt-sensitive type. *TPI* was downregulated in the salt-tolerant oat but upregulated in the salt-sensitive type, and *HXK6* was downregulated in both oat types. *FBA* expression was gradually reduced after 6 h of stress in the salt-tolerant type. *HXK6* expression gradually reduced in the salt-tolerant and -sensitive types. Further, after 12 h, the expression of *ATP-PEK*, which is related to pentose phosphate, and *TKT,* was upregulated in the salt-tolerant type. In the salt-sensitive type, *ATP-PEK* was upregulated, but *TKT* was downregulated (Figure 7). The results demonstrated that the expression trends observed in the RNA-seq data and transcriptome analysis were largely consistent. The RT-qPCR results were consistent with the sequencing results, thus validating the accuracy of the transcriptome sequencing data.

## 4. Discussion

### 4.1. Glycolysis

Studies on wheat seeds [22], watermelons [23], and sweet potatoes [24] under abiotic stress have shown that an increase in soluble sugars helps plants resist abiotic stress. In this study, the soluble sugar content of both varieties of oats gradually increased; however, at 48 h, the increase in the sugar content in response to salt–alkali stress in Menglong oats from 24 to 48 h was slightly greater than that in Qinqiong oats, but the content of soluble sugars in Qinqiong oats was still substantially greater than that in Menglong oats. There were also significant differences in the transcriptional level expressions between the two varieties of oats. Glycolysis is an important metabolic pathway in the salt stress response that enhances osmotic tolerance and increases antioxidant activity [25]. GAPDH is a key enzyme in the glycolytic metabolic pathway that plays a crucial role in stress resistance in organisms [26]. In the tolerant type, 47 *GAPDH* genes were significantly upregulated, while in the sensitive type, 11 *GAPDH1* genes were partially and significantly upregulated under saline–alkali stress. *FBP* plays a role in the antioxidant defence of pea leaves, exhibiting a potent capacity to scavenge superoxide anions. This suggests that *FBP* contributes to a non-enzymatic antioxidant mechanism in plants experiencing cold stress [27]. In the tolerant type, twelve *FBP* genes were significantly upregulated, whereas, in the sensitive type, three *FBP* genes were partially and significantly upregulated under saline–alkali stress. *iPGAM* facilitates the reversible transformation between 3-phosphoglycerate and 2-phosphoglycerate within the glycolytic pathway [28]. Two *iPGAM* genes in the tolerant type were upregulated at all time points except 48 h, whereas nine *iPGAM* genes were significantly upregulated in the sensitive type under saline–alkali stress. Heterologous expression of *PhPGK1* and *PhPGK2* in transgenic *Chlamydomonas reinhardtii* significantly enhances the tolerance of the algae to high temperatures [29]. Under saline–alkali stress, six *PGK* genes in the tolerant type and five in the sensitive type were significantly upregulated. *PK* is a key regulatory enzyme in the glycolytic metabolic pathway that is involved in abiotic stress responses [30]. Under saline–alkali stress, fifteen *PK1* genes in the tolerant type and eight in the sensitive type were significantly upregulated. The transcription level of *OscTPI* responds to various abiotic stressors [31]. In the tolerant type, seven *TPI* genes were downregulated before 24 h and upregulated at 48 h, whereas two *TPI* genes were upregulated in the sensitive type at all four time points under saline–alkali stress. Cai et al. found that under conditions of heat and cold stress, *FBAs* in tomato seedlings were significantly expressed [32]. In the salt-tolerant variety, 155 *FBA* genes were downregulated within 6 h, and the number of *FBA* genes gradually decreased with increasing treatment duration. In contrast, 12 *FBA1* genes were partially downregulated in the salt-sensitive type under saline–alkali stress.

### 4.2. Pentose Phosphate Pathway

The pentose phosphate pathway is an important respiratory pathway in plants and is enhanced under stress conditions such as low temperature and drought [33]. Under salt stress, fructose levels in Arabidopsis mutants are lower [34]. The primary function of *bfFBA* is to break down fructose. In this study, 154 genes were significantly upregulated in the salt-tolerant type, whereas 34 genes were significantly upregulated in the salt-sensitive type. Studies on both salt-sensitive and salt-tolerant rice varieties have indicated that *FBP* activity decreases during growth under salt conditions in salt-sensitive rice [35]. In this study, the expressions of twelve genes were significantly upregulated at 48 h in the salt-tolerant type of oat, and those of two genes were significantly upregulated at 6 h in the salt-sensitive type of oat but significantly downregulated at 12 h. Studies have shown that drought and bicarbonate stress inhibit *PFK* activity in Aloe vera leaves [36]. In this study, *ATP-PFK* expression was continuously downregulated in the salt-tolerant type of oat, with the most significant downregulation at 48 h, whereas in the salt-sensitive type of oat, the expressions of three genes were mostly upregulated at four different time points. Pyrophosphate regulates the response of plants to salt and alkaline stress by adjusting the activity of the H^+^-ATPase in the vacuoles of barley roots [37]. *PFP* mainly participates in the breakdown of fructose, and in this study, 13 *PFP* genes were only significantly upregulated at 6 and 12 h under salt and alkaline stress in the salt-tolerant type, while no significant expression was found in the salt-sensitive type. Studies on chickpeas indicate that the transcription level of *NADP-GAPDH* decreases under the influence of water stress [38]. In the present study, *NADP-GAPDH* was only significantly upregulated at 48 h in the salt-tolerant type. Four *PGLS* genes were present only in the salt-tolerant type, and all were upregulated at 24 h. The activity of transketolase is moderately increased under salt conditions in maize seedlings [39]. In this study, 17 *TKT* genes were upregulated in the salt-tolerant type, and this upregulation continued to increase. *TAL* is a key enzyme in the pentose phosphate pathway, and studies on *Arabidopsis*, rice, and other plants have found that this gene has an impact on vascular formation, lignin synthesis, and plant stress resistance [40]. Four *TAL* genes were downregulated in the salt-tolerant type, with significant downregulation observed at 48 h.

### 4.3. Starch and Sucrose Metabolism

Under saline–alkali stress, studies on rice have found that the starch content in rice roots gradually decreases [41]. The starch content in Menglong oats gradually decreased, whereas it was relatively stable in Qinqiong oats. Based on the transcriptome data, it is very likely that the differential expression of genes related to starch expression in two varieties of oats occurs after saline–alkali stress. Previous studies have shown that plants respond to stress by maintaining a normal physiological metabolism [42]. Studies have suggested that starch synthase (SS) is a key regulatory enzyme in starch biosynthesis. The expression patterns of *SS* genes under drought conditions are correlated with SS activity and starch content in developing grains [43]. In the present study, five *SS* genes were exclusively expressed in the salt-tolerant oat variety, with most exhibiting significant downregulation at 6 and 12 h. In cabbage, *glgB* transcript levels gradually increased under drought stress [44]. The current study identified 21 *glgB* genes expressed solely in saline-tolerant oats, which were significantly downregulated during all four time periods. Research on jujube has revealed that the various expression patterns of the *ZjBAM* gene family members suggest their critical roles in jujube growth, development, and abiotic stress responses [45]. In this study, twelve *BAM* genes in the saline-tolerant varieties were mostly downregulated during the four time periods, and five *BAM* genes in the saline-sensitive varieties were significantly upregulated at 6 and 12 h and predominantly downregulated at 24 and 48 h. In studies of cassava, *Me AMY3* was identified as a key gene in the response to drought stress based on its expression pattern under drought conditions [46]. In the present study, two *AMY3* genes were expressed only in saline-tolerant varieties, with predominantly upregulated expression in the four time periods.

Sucrose acts as a long-distance signal that is sent in increased concentrations from the shoot to the root in response to various nutrient deficiencies [47]. After exposure to salt–alkali stress, the sucrose content in Qinqiong (salt–alkali-resistant oat) was more than twice that of Menglong (salt–alkali-sensitive oat) before 48 h. There are also significant differences in the transcriptional level expressions between the two varieties of oats. Sucrose participates in plant stress responses and is a protective feedback mechanism in response to stressful environments [48]. Increased *SPP* activity in seedling leaves under sodium chloride treatment alleviated this stress effect [49]. In the present study, under salinity stress, three *SPP* genes were expressed solely in saline-tolerant oat varieties and were predominantly upregulated, except at 6 h, during the four time periods. Related studies have reported that drought stress induces *HvSUS3* expression in barley [50]. In this study, sixty-six genes were significantly downregulated in saline-tolerant oats at 12 and 48 h, and six genes were significantly upregulated at 6 and 48 h. Previous studies have shown that regulation of SL (xylipin sheet) deposition is one of the mechanisms by which *AtTPS9* imparts salt tolerance to Arabidopsis plants [51]. In the present study, eighteen *TPS* genes were significantly downregulated in saline-tolerant oats during the four time periods, and four *TPS* genes were downregulated in saline-sensitive oats at 12 and 48 h. Studies on tobacco have suggested that the *NtINV* gene is involved in tobacco leaf development and tolerance to environmental stress [52]. In the present study, 33 *INV* genes were expressed only in saline-tolerant varieties and were downregulated, except at 12 h, for the four time periods.

### 4.4. Fructose and Mannose Metabolism

Studies on potato tubers showed that drought and waterlogging increased levels of fructose, glucose, and sucrose in most potato cultivars [53]. In this study, the fructose content in both oat varieties increased gradually; however, after exposure to stress, the fructose content in Qinqiong oats was higher than that in Menglong oats. Based on transcriptome data, it is very likely that the expression of genes related to fructose expression in the two varieties of oats occurs after saline–alkali stress. Fructose is a key signalling molecule that regulates plant growth, development, and defence [54]. Mannose, an important monosaccharide, is involved in coping with abiotic stresses in plants [55]. Previous studies have shown that mannose can induce the accumulation of multiple sugars (e.g., soluble sugars and glucose) and sugar alcohols under drought conditions, which not only improves cell osmoregulation ability under drought stress but also provides more available carbohydrates for metabolic energy supply [56]. Two *GMD 2* were identified in tolerant oats and exhibited a downregulation trend under the four stress durations, whereas one *GMD 2* was observed in susceptible oats, which demonstrated upregulation at 24 h and downregulation at 48 h. Fru-2,6-P 2 is a signalling molecule that controls glycolysis. *PFK-2* is a homodimeric bifunctional enzyme that catalyses the synthesis and degradation of Fru-2,6-P2 [57]. In the present study, only seven differential *PFK-2* genes were identified in tolerant oats, which were upregulated at all four stress durations, with the increasing ratio demonstrating a gradual increase. *HXK* is a bifunctional enzyme involved in sensing carbohydrates and sugar signalling. *HXK* affects plant growth and development in response to nutrient availability [58]. In the *HXK* gene family, salt-tolerant oats significantly express *HXK2*, *HXK3*, *HXK5*, *HXK6*, *HXK7*, and *HXK8*. However, only *HXK5* was significantly expressed in salt-sensitive oats. *MdPFK5* overexpression confers salt tolerance in apple callus and Arabidopsis transgenic lines [9]. *PFK* is significantly expressed in tolerant varieties, including *PFK2*, *PFK3*, and *PFK5*. However, only *PFK2* and *PFK3* were significantly expressed in sensitive varieties. *FRK* plays a key role in carbon allocation, plant development, and abiotic stress responses [59]. The expressions of *FRK1* and *FRK2* were found to be upregulated under the four stress durations in the tolerant varieties. *SDH* plays a role in plant tolerance to salt and osmotic stress [60]. In this study, *SDH* was significantly expressed only in salt-tolerant oats and was downregulated under the four stress durations. Chen et al. demonstrated that the loss of *SITPI1* and *SITPI2* significantly affects photosystem proteins and diminishes photosynthetic capacity, whereas *SITPI1* and *SITPI2* exhibit potential applications in enhancing heat resistance in crops [61]. In this study, *TPI1* was only expressed in salt-tolerant oats and was upregulated under the four stress durations.

## 5. Conclusions

**Figure 8:**
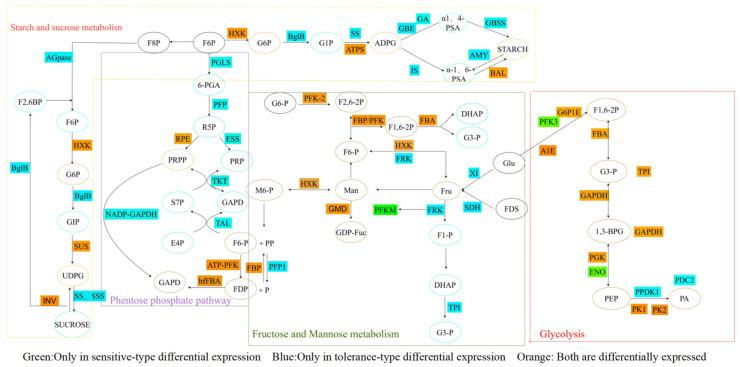
The regulatory mechanisms of differentially expressed genes in Menglong Yannai (stress-sensitive) and Qinqiong Yannai (stress-tolerant) oats under salt–alkaline stress in the processes of glycolysis, pentose phosphate pathway, starch, sucrose, fructose, and mannose metabolism. (Using *p* < 0.05, |FC| ≥ 2 as the screening criteria, Green: Only in sensitive-type differential expression; Blue: Only in tolerance-type differential expression; Orange: Both are differentially expressed).

The transcriptional profiling of 30 saline–alkaline-tolerant and saline–alkaline-sensitive oat samples was found to be reliable and consistent. Comparative transcriptomic analysis between the two oat varieties identified 1707 and 1328 significant DEGs related to sugar metabolism under saline–alkaline stress, respectively. Upon 6, 12, 24, and 48 h of stress, 197, 52, 85, and 254 shared significant DEGs were detected in both varieties. Further annotation using KEGG for carbohydrate metabolism pathways revealed enrichment in glycolysis, the pentose phosphate pathway, and starch, sucrose, galactose, and mannose metabolism. Detailed discussion and RT-qPCR validation of the significant DEGs in these pathways further confirmed the expression of genes such as *SUS2*, *GADPH*, *PPDK1*, *TPI*, *HXK6*, *FBA*, *ATP-PEK*, and *TKT*. Finally, we integrated the expression profiles of the significant DEGs across four pathways related to sugar metabolism and crafted a mechanistic map of these pathways under salt–alkaline stress. This allows for a clear revelation of the regulatory mechanisms of sugar signalling under such stress conditions (Figure 8).

## Figures and Tables

**Table 1:** Primers used for gene expression analysis.

Gene Name	Forward Sequences (5′-3′)	Reverse Sequences (5′-3′)
*FBA4*	GCTCTAGAAGCACACACACTGTTAGC	GCGGTACCCTGGCTAACAAAGAGGAA
*GAPDH*	ATGTTCAAATATGACACCGTTC	TCGGGATTTCTAGCACCA
*TPI*	TGATGTTCGTGCTTATCTTTCC	GTCAGGTTGAGTGGCAAGTTC
*SUS2*	ATGGCTGATCACAGAACCTTGA	TTAATCATGGTGCAAAGGAAC
*ATP-PFK*	TGCCCTGGTCTCAATGATGTCA	TGATAAGCACTATGTGCCTCAAT
*TKT*	ATAAGGTACCGAGCTCGGATCCTAAGGGTAAACACATAAGGA	ATAAGGTACCGAGCTCGGATCCTAAGGGTAAACACATAAGGA
*HXK6*	TGTGACATAGTGACGGAGCG	AGGAACAAAGCACCAGTTCCA
*ISA3*	GATTGGACTCGAGCATTTGTGGTAG	GCTTCTCAGGAGTTCAAGCAGATGG
*PPDK1*	CCGCTCGAGATGGCGGCATCGGTTTCC	CGGGATCCTGACAAGCACCTGAGCTG
*BAM2*	TGTGAATAGAAAGAAGGCGATG	CTTGGGTAAAGGGATAGAGACG
*Actin*	GATGCTGAGGATATTCAACCCC	CCATGACACCAGTATGACGAGG

## Data Availability

Data will be made available on request. The raw RNA-seq data (Accession no.PRJNA1087546) were uploaded to NCBI.
